# HRTEM analysis of the aggregate structure and ultrafine microporous characteristics of Xinjiang Zhundong coal under heat treatment

**DOI:** 10.1038/s41598-022-09113-z

**Published:** 2022-03-23

**Authors:** Xueping Li, Qiang Zeng

**Affiliations:** 1 College of Resource and Environmental Sciences, Xinjiang University, Urumqi, 830046 China; 2 Institute of Arid Ecology and Environment, Xinjiang University, Urumqi, 830046 China; 3 Key Laboratory of Oasis Ecology of Ministry of Education, Xinjiang University, Urumqi, 830046 China

**Keywords:** Coal, Energy

## Abstract

Understanding the change in coal structure during heat treatment is the basis of efficient and clean utilization of coal. In this study, high-resolution transmission electron microscopy (HRTEM) was used to analyse the changes in the aggregate structure and ultramicropores of Zhungdong coal samples (Xinjiang, China) that were heated from ambient temperature to 800 °C respectively. Then, the relationship between their HRTEM characteristics and the corresponding reaction activation energy were also analyzed. The results show that the length, curvature, order, layer spacing and stacking height of the aromatic layers of the coal sample vary with an increasing temperature, and are related to the activation energy of the reaction. As the temperature reaches 300 °C, the HRTEM characteristics of the heated coal samples are obviously different from those of the raw coal sample. It is shown that the length of lattice fringes is in the range of 0.3–1.15 nm which accounts for approximately 95% of the total number of fringes. The overall orientation of lattice fringes is not good, but there are two main directions. After heating, the number of naphthalenes in the coal samples decreased, while the number of larger aromatic layers increased. The distance between the aromatic layers of the coal sample decreased with an increasing stacking height, the order of the aromatic layers was enhanced, and the number of aromatic sheets with a larger curvature increased. The coal ultramicropores are mainly concentrated from 0.4 to 0.7 nm. Heat treatment reduces the total number of ultramicropores, but the maximum number of pores is increased. The non-six-membered ring and lattice defects lead to the bending of the fringes, the distribution of fatty structures affects the orientation of the fringes, and the relationship between the pore and molecular structure does not exist independently. After heat treatment, the aggregate structure and ultramicropore size of coal have a high correlation with the activation energy. The activation energy is closely related to the 0.6 nmultramicropores. However, the current experiment could not explain the underlying causes of these relationships. The aggregated state in coal is the macromolecular group formed between different aromatic structures, fat structures and other molecules, which is formed by the interaction of internal defects and pores in the molecular group. The structural differences at different temperatures therefore reflect the interaction of different macromolecules in coal.

## Introduction

The study of coal structure involves understanding and the rational utilization (gasification, combustion, coking etc.) of coal resources^1^. It is also is one possible way to quantify the characteristics of coal oxidation and spontaneous combustion. In addition, the treatment is helpful for understanding the structural changes of coal during spontaneous combustion and conversion^2^. The structure of coal is characterized by coal seam stacking and the distribution characteristics of aromatic lamellae^3^. In addition, high-resolution transmission electron microscopy (HRTEM) was applied to the layer structure of carbon materials in the 1960s. With the continuous update of the HRTEM test method, it has been widely used in studies on coal structure thus far. HRTEM can detect the lattice fringes of coal and provide direct information about the structure of coal at the atomic level. Using HRTEM image processing technology, the length and orientation of the coal aromatic layer can be directly expressed. HRTEM not only has a high resolution, but is also a valid diffraction device, that can be used to directly observe the morphological characteristics of a substance while also obtaining a large amount of microcrystalline structural information.

Sharma et al.^4^ reported for the first-time clear images of TEM lattice fringes of coal and observed that the entire structure was amorphous except for some orientations of fringes outside the edges. Sharma et al.^5–8^ quantitatively studied the fringe length and the number of stacking fringes of Argonne Premium raw coal, and revealed that with the deepening of coal metamorphism, the average length and the average number of stacking of the aromatic layers were obtained. Since then, many scholars have improved the method of HRTEM image feature extraction. Davis et al.^9^ developed a quantitative analysis method that uses the Fourier Transform (FT) to filter the original image to eliminate the aperiodic structure. From this, they obtained the average fringe length and the relative content of the crystal structure. Palotás et al.^10^ used HRTEM and image analysis technology to systematically study the structural changes of soot and carbon black particles during the combustion process. The method also used the FT of HRTEM images to extract the structural parameters, and then performed an inverse transformation. Wornat et al.^11^ used HRTEM to analyse the structural transformation of biochar during combustion.

HRTEM can also obtain coal curvature information, where the orientation increases the reactivity of coal^12^. Wang et al.^13^ using MATLAB code written by Nottingham University, proposed an inflection point, angle, and segment length analysis method that can identify the string of the bending lattice. This method is identified by identifying inflection points, segments, segment length, segmentation, and accumulation angles of each segment. Gao et al.^14^ updated the HRTEM image processing method and determined the average microcrystalline diameter, non-graphite amorphous index, orientation sequence parameter number and bending degree. Observing from the HRTEM image of different ranks of coal, the lattice stripes have a significant curvature, with an increase in the coal grade, the overall curvature of the lattice stripes decrease, and irregular lattice stripe rules are observed^15^. However, many scholars are currently characterized by HRTEM to characterize the coal structure only on the measurement of the extraction and length of their lattice stripes, with less research on the characterization of orientation. This is because there is an unresolved technical problem in the quantization of curvature.

Recently, Endo et al.^16^ used HRTEM combined with image processing software to observe and study the pores in activated carbon fiber. Pan et al.^17^ used the image analysis algorithm to perform a quantitative analysis on HRTEM images of different types of tectonic deformed coals to obtain pore structure parameters (pore width, pore area and pore length). The results show that the coal-stage has an important impact on the ultra-micro holes of the coal macromolecular structure, which vary with the variation phase. When the structure deformation is weak, as the coal stage increases, the width of the ultramicrohole is reduced, and the length and hole area of the ultramicrohole are increased. Studies using HRTEM images of coal pores need to be further developed.

In general, with an increasing maturity of image processing technology, the HRTEM study of coal has been qualitatively analysed to a new stage of quantitative characterization. Although many scholars have made a characteristic of qualitative to quantitative structures on the aggregation state of different coals, there are few studies that classify the structure of the aggregation structure in coal, and less research on the orientation and ultramicropores.

In this paper, HRTEM images of coal samples treated at different temperatures are processed, and the length, orientation, curvature and stack structure characteristics of lattice fringes are extracted via image processing technology. The ultramicropore information of the HRTEM image is obtained using the watershed algorithm. In addition, a comparison of the relationship between the activation energy and reaction parameter aggregation structure and ultramicropore characteristics was performed. At the same time, the change in the aggregated structure of coal under different heat treatment conditions is discussed, and the differences in the lattice fringes and the complexity of the structure are revealed, in order to provide the basis for achieving green sustainable development of coal resources in the Zhundong mining area.

## Samples and methods

### Samples

Coal samples were collected from the Bm coal seam of the Wucaiwan mining area, eastern Junggar, Xinjiang, in accordance with international (GB/T482-2008) collection principles^18^. The coal sample was crushed and ground to below 200 mesh, then acid treated for deashing and vacuum dried for reserve use.

Zhundong coal has rich reserves in China, with a high moisture and volatile content, ash, and low heat value. However, it is easy to fire, and is flammable. The iron, potassium and sodium contents of the coal ash are high, resulting in a decrease in the ash melting point, which easily generates a slag. This can cause a high temperature heat exchange and reaction equipment to be stained.

### HRTEM image analysis

High-resolution transmission electron microscopy (JEM-2010) made by Japan Electronics Co., Ltd was used for the observation. The acceleration voltage was 200 kV, the point resolution was 0.19 nm, and the lattice resolution was 0.14 nm. Each sample was diluted with ethanol and was homogenized with ultrasonic energy for 20 min to aid the dispersion. Then the mixture of the sample and ethanol was sprayed over a silicon nitride film. The samples were heated in situ from the temperature range of ambient temperature to 800 °C at a rate of 1000 °C per second and were examined every 50 °C to observe their structural changes with an increasing temperature. HRTEM images were obtained from the different points of each sample. To enhance the representativeness of the data, more than ten points were taken for processing the data at each temperature stage. To obtain quantitative information on these parameters, the lattice fringe images were analysed as follows.

#### Image preprocessing

To obtain a clear image of graphite microcrystalline fringes for calculating the aggregation state parameters, it is necessary to denoise the original image first, then set a reasonable threshold to extract the lattice fringes, and finally convert the lattice fringes into vector data (vector lines)^19^. This process is defined as the image preprocessing process, which is realized through MATLAB programming^20^. Procedures: original-FFT-Filter-IFFT-Binarization-Skeletonization-Vectorization-Lattice fringe.

**Figure 1:**
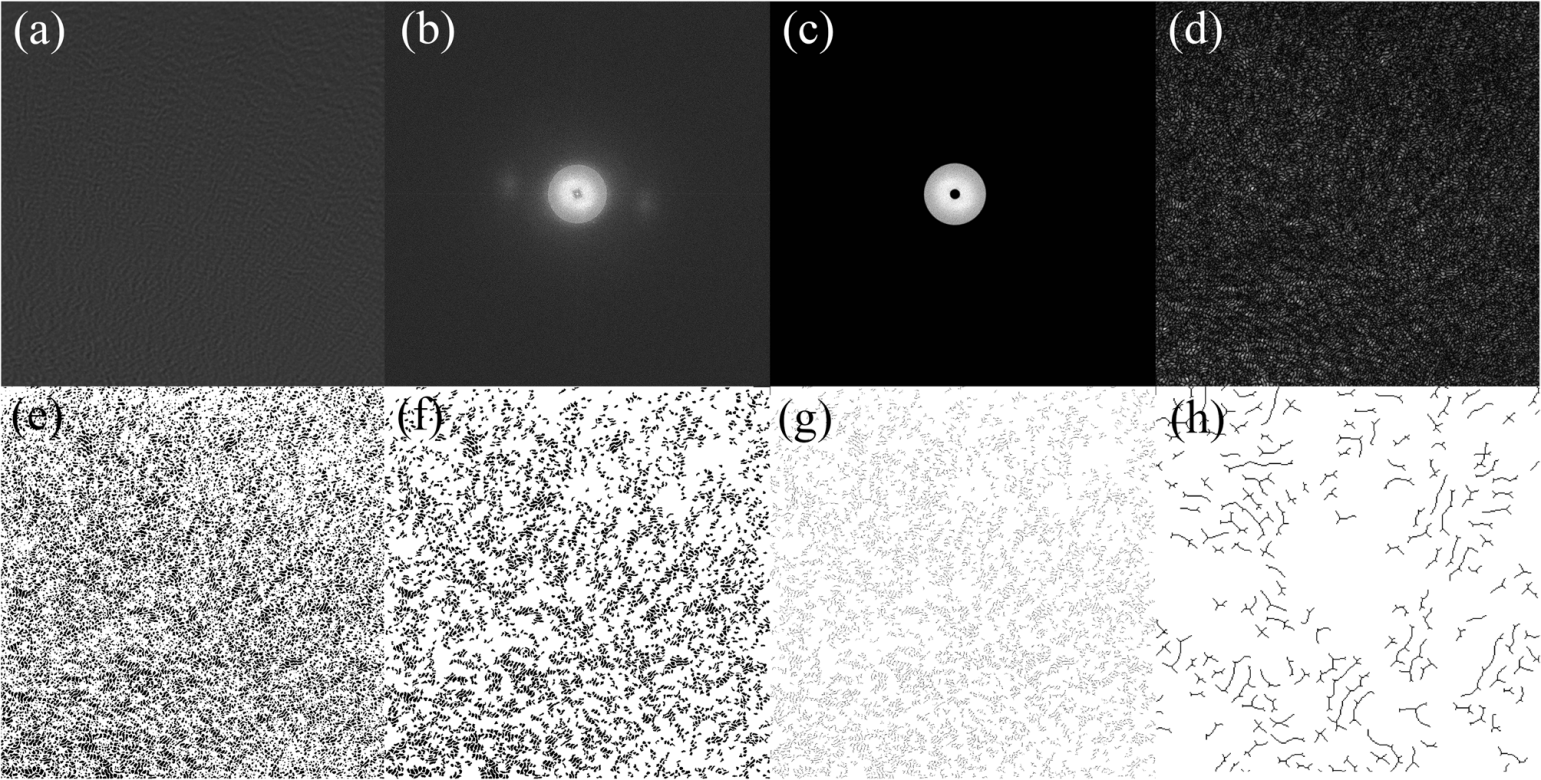
Diagram of image processing on lattice image of coal (**a**) Original image, (**b**) FFT image, (**c**) Loop filter, (**d**) IFFT image, (**e**) Figure of Binarization, (**f**) Figure of Skeletonization, (**g**) Figure of Vectorization, (**h**) Partial enlarged detail of Figure vector quantization.

The specific content and purpose of preprocessing are as follows: first, image noise reduction is conducted. Diffraction spots in the original image (Fig. 1a), namely microcrystalline fringes, filter the disordered part in the coal structure to realize periodic information enhancement and make the lattice fringes clearer. The original HRTEM image was transformed by FFT (Fig. 1b) to obtain the frequency domain image, and then the frequency domain image was filtered using a ring in a certain frequency range (Fig. 1c), Then the IFFT transform was performed (Fig. 1d). The second is image binarization (Fig. 1e) to separate the microcrystalline fringe from the background information. The pixel grey value of each point in the image is compared with the threshold value. If the pixel grey value is greater than the threshold value, then the pixel will be assigned a grey value of 1, and the point will be displayed as white, that is the image background. If the pixel grey value is less than the threshold value, the pixel will be assigned a grey value of 0, and the point will be displayed as black, that is the lattice stripes. The third is skeletonization (Fig. 1f), which converts the stripes in the figure into single pixel stripes. After binarization, the microcrystalline fringes have a certain thickness. The thickness of two-dimensional graphite lamellae can be ignored. After erosion, they are expanded and then skeletonized into single pixel thickness fringes, where the noise of the lattice length less than 0.3 nm is removed. The fourth is vectorization (Fig. 1g,h), with trimming to extract the fringe skeleton. The connected aromatic layers are separated and line vectorization is performed according to the principle of drawing long lines.

#### Aggregate structural parameters of coal

In this study, some characteristics derived from HRTEM were used to discuss the chemical structural changes with heat-treatment, including the fringe length, orientation, degree of curve, and the stack structure. The distributions are the average of the distributions obtained from at least 10–15 fields for each sample.

**Figure 2:**
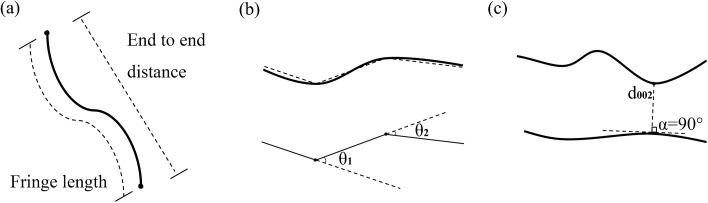
Illustrations for calculating the (**a**) fringe length and tortuosity, (**b**) degree of curve (**c**) interlayer spacing (d_002_).

1. Fringe length.
  The length of the HRTEM lattice fringe represents the physical length of the aromatic layer fragment, which is obtained by the length of the vector line (Fig. 2a). According to the classification method of Niekerk D V and Mathews J P (Table 1)^21^, lattice fringes of different lengths can be divided into different attributions.

**Table 1:** Classification of HRTEM lattice fringes.

Aromatic sheet	Mean (d/nm)	Grouping (d/nm)
1 × 1	0.39	0.30–0.54
2 × 2	0.60	0.55–0.74
3 × 3	0.93	0.75–1.14
4 × 4	1.27	1.15–1.44
5 × 5	1.60	1.45–1.74
6 × 6	1.94	1.75–2.04
7 × 7	2.28	2.05–2.44
8 × 8	2.61	2.45–2.84
2. Orientation.
  Orientation can reflect the order degree of the arrangement of aromatic layers. Orientation is calculated using the linear directional mean (LDM) of different regions of the fringe^13^. Formulae (1) and (2) are used to adjust the extra quadrant of the calculated angle to obtain the direction distribution of the lattice fringe.
  1 $$LDM = \tan^{ - 1} \frac{{\sum\limits_{i = 1}^{n} {{\text{Sin}} \theta_{i} } }}{{\sum\limits_{i = 1}^{n} {{\text{Cos}} \theta_{i} } }}$$
  2 $$f\left( {\ddot{x}} \right) = \left\{ {\begin{array}{*{20}l} {LDM} \hfill & {\sum\limits_{i = 1}^{n} {{\text{Sin}} \theta_{i} } \ge 0} \hfill & {\sum\limits_{i = 1}^{n} {{\text{Cos}} \theta_{i} } > 0} \hfill \\ {180^{^\circ } - LDM} \hfill & {\sum\limits_{i = 1}^{n} {{\text{Sin}} \theta_{i} } \ge 0} \hfill & {\sum\limits_{i = 1}^{n} {{\text{Cos}} \theta_{i} } < 0} \hfill \\ {360^{^\circ } - LDM} \hfill & {\sum\limits_{i = 1}^{n} {{\text{Sin}} \theta_{i} } < 0} \hfill & {\sum\limits_{i = 1}^{n} {{\text{Cos}} \theta_{i} } > 0} \hfill \\ {180^{^\circ } + LDM} \hfill & {\sum\limits_{i = 1}^{n} {{\text{Sin}} \theta_{i} } < 0} \hfill & {\sum\limits_{i = 1}^{n} {{\text{Cos}} \theta_{i} } < 0} \hfill \\ \end{array} } \right\}$$
  where LDM is the linear directional mean of different regions of the fringe, θ_i_ is the direction of a group of broken line elements starting from a single source, and n is the total number of elements.
3. Degree of the curve.
  The degree of the curve reflects the horizontal fluctuation or nonlinearity of a single aromatic layer, including three indices: average tortuosity, segment angle and cumulative angle, where, the average tortuosity is the ratio of the fringe length to the end-to-end distance (Fig. 2a)^22^, which represents the linear condition of the entire aromatic layer fragments in the HRTEM images. In other words, the smaller the average curvature is, the better the integrity of the aromatic layer^23^. The segment angle represents the change in the angle between each fringe segment. In Fig. 2, a single fringe in b contains two segment angles, θ_1_ and θ_2_. The overall bending of the lattice fringe can therefore be reflected by calculating the angle changes between all the segments of the fringe. The cumulative angle represents the sum of the angle changes between individual fringe segments. The cumulative angle of the stripes in Fig. 2 is the sum of θ_1_ and θ_2_. By calculating the sum of the segment angles of each fringe, the bending situation of a single lattice fringe can be reflected, thus obtaining the cumulative angle sizes of lattice stripes with different lengths^24^.
4. Stack structure.
  The stack structure of coal is analysed from HRTEM, including the interlayer spacing (d_002_), the layer size (La), the aromatic layer stacking height (Lc), and the number of aromatic layers per stack (n)^25^. Interlayer spacing (d_002_) is defined as the average distance between adjacent parallel stripes, which is formed by manually selecting approximately parallel adjacent stripes, and is the minimum distance of a pair of stripes (Fig. 2c). The layer size (La) is calculated by weighting of the lattice fringe length. Since there is no good parallelism between the stripes, most of the stripes show the characteristics of distribution in different directions, so the stacking height (Lc) and l the number of aromatic layers per stack (n) in this study are randomly selected for the measurement of orderly stripes in the 3 nm × 3 nm region. The distance between the effective nearly parallel stripes in the region is the stacking height (Lc), and the number of stripes is the number of aromatic layers per stack (n)^26^.

#### Characterization of the ultramicropores

The gap between adjacent aromatic layers in the macromolecular structure of coal is called the ultramicropores (< 1.1 nm) due to incompact stacking^27,28^. For HRTEM image denoising and binarization inversion, a pixel greyscale value larger than the threshold value is converted to 255, and this point is displayed as black, which is a possible ultrafine pore; a pixel greyscale value less than the threshold value is converted to 0, and this point is displayed as white, which is the background. A watershed segmentation algorithm is used for the inverted image, and the obtained patches are ultramicropores^29^.

### Thermogravimetric analysis

The coal sample was heat treated using a muffle furnace, and each temperature was allowed to stand at 100 °C for 10 min. Then the sample was removed until the temperature was warmed to 800 °C. Thermogravimetric analyses were performed using a HITACHISTA 7300 thermal analyser from Japan The experimental conditions were as follows: the heating rate was10 °C/min, the atmosphere was air, the air flow rate was 200 mL/min, and the control temperature was raised from ambient temperature to 1000 °C^30^. Based on thermogravimetric data and Arrhenius law, the Coats-Redfern integral method was used to calculate the activation energy^31^, using Formulas (3) and (4).

3 $$n = 1,\ln \left[ {\frac{{ - \ln \left( {1 - \partial } \right)}}{{T^{2} }}} \right] = \ln \left[ {\frac{AR}{{\beta E}}\left( {1 - \frac{2RT}{E}} \right)} \right] - \frac{E}{RT}$$

4 $$n \ne 1,\ln \left[ {\frac{{1 - \left( {1 - \partial } \right)^{1 - n} }}{{T^{2} \left( {1 - n} \right)}}} \right] = \ln \left[ {\frac{AR}{{\beta E}}\left( {1 - \frac{2RT}{E}} \right)} \right] - \frac{E}{RT}$$

where n is the order of reaction, α is the mass conversion rate, β is a constant heating rate (K/min), A is the pre-exponential factor, E is the activation energy (KJ/mol), T is the reaction temperature (K), and R is the molar gas constant, where R ≈ 8.3145 J/(mol K).

The coal oxygen combustion reaction is considered as a primary reaction, and E/RT ≥ 1, 1-2RT/E is approximately to 1, which is simplified by Formula (3) into Formula (5).

5 $$n = 1,\ln \left[ {\frac{{ - \ln \left( {1 - \alpha } \right)}}{{T^{2} }}} \right] = - \frac{E}{RT}$$

With ln[− ln(1 − α)/T^2^] as the ordinate, − 1/T is a horizontal synchronization method to obtain a straight line, and the slope of the straight line represents − E/R, thereby obtaining the activation energy of the reaction.

## Results and discussion

### Length distribution of the aromatic layer

**Figure 3:**
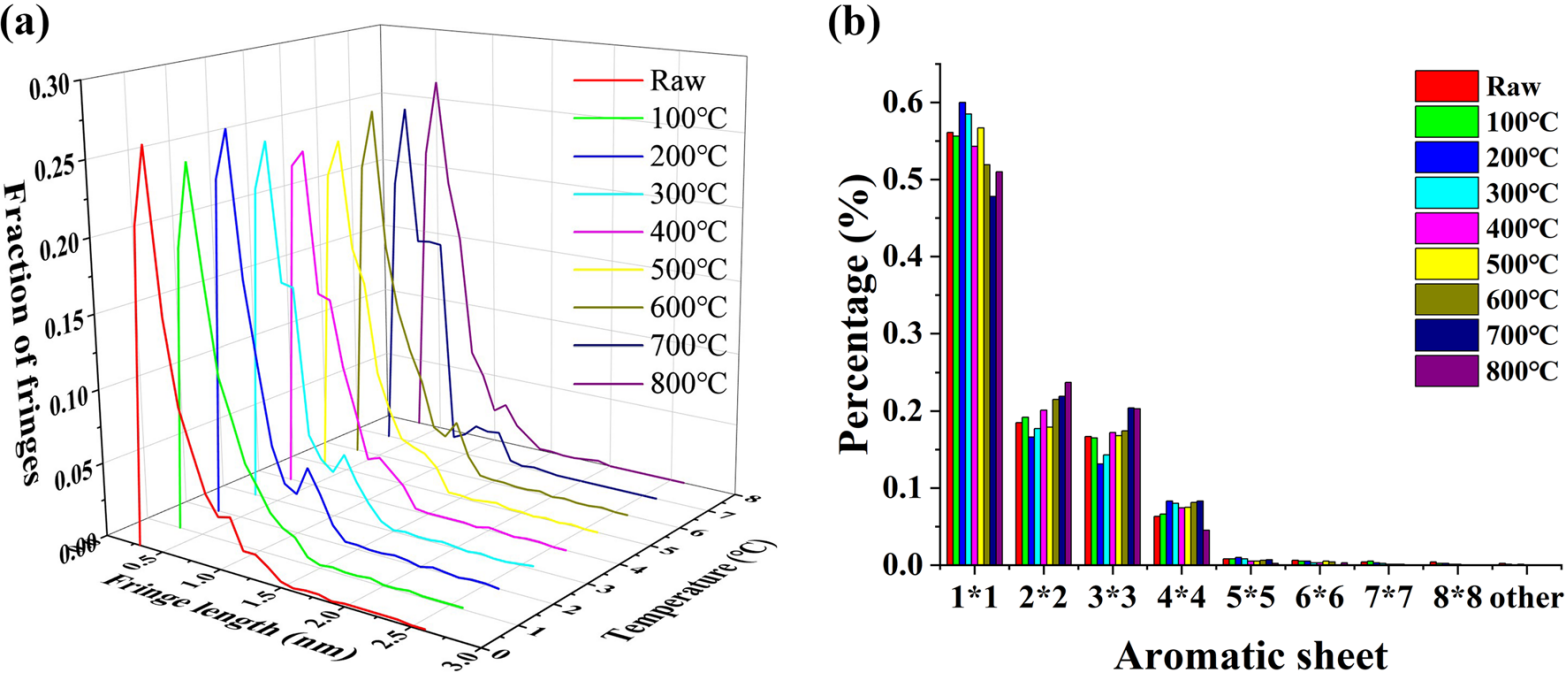
(**a**) Fringe length frequency and (**b**) different aromatic structures of the content distribution at different temperatures.

After extraction, the length of the lattice fringes ranges from 0.3 to 3.0 nm, and the coal samples treated at different temperatures have similar variation trends, with peak values at 0.5 nm and 1.1 nm, indicating a rapid increase (Fig. 3a). According to the classification method of Niekerk and Mathews (Table 1)^21^, the most significant feature is the rich content of naphthalene (1 × 1), followed by the content of 2 × 2 and 3 × 3 aromatic layers, which account for 95% (Table 2).

**Table 2:** Percentage of different aromatic sheets at different temperatures.

	Raw (%)	100 °C (%)	200 °C (%)	300 °C (%)	400 °C (%)	500 °C (%)	600 °C (%)	700 °C (%)	800 °C (%)
1 × 1	56.1	55.6	60.0	58.5	54.3	56.7	51.9	47.8	51.0
2 × 2	18.5	19.2	16.6	17.7	20.1	17.9	21.5	21.9	23.7
3 × 3	16.7	16.5	13.1	14.3	17.2	16.8	17.4	20.4	20.3
4 × 4	6.3	6.6	8.3	8.0	7.4	7.5	8.1	8.3	4.5
Amount	97.6	97.9	98.0	98.5	99.0	98.9	98.9	98.4	99.5

In addition, based on the coal pyrolysis process and the change of naphthalene content with temperature, the change of naphthalene can be divided into four stages (Fig. 3b and Table 2). The first stage was at ambient temperature to 200 °C, where the naphthalene content was increased and reached the peak (60.0%), which may be due to a large number of carboxyl groups and oxygen-containing functional groups from aromatic structures. The second stage is from 200 to 400 °C where the content of naphthalene decreased significantly (54.3%), probably as a result of the pyrolysis of aliphatic hydrocarbons and hydroxyl groups on the aromatic rings. The third stage is from 400 to 600 °C, with an increasing temperature, more stable ethers and oxygen-containing heterocycles also began to be pyrolyzed. At approximately 500 °C, the naphthalene content increases again (56.7%). The fourth stage is lowered from 600 to 800 °C, and the content of naphthalene was reduced to approximately 50%, which may be aromatic layer polymerization. Therefore, in the test temperature range, the two temperature ranges with higher naphthalene contents were concentrated at 200 °C and 500 °C^32^. Compared with the trend of the naphthalene content, the content of 2 × 2, 3 × 3 and 4 × 4 aromatic layers increased during the heating process.

The change in the 1 × 1 aromatic layer in the low temperature stage may be attributed to the decomposition of oxygen-containing functional groups such as carboxyl groups. These functional groups are often connected with aromatic structures or exist in the form of cross-linking structures. With an increasing of temperature, they decompose into gaseous products and lead to the formation of some small aromatic layer sheets^33^. In addition, for stable oxygen-containing heterocycles and ether bonds and other oxygen-containing structures, oxygen-containing heterocycles and aromatic rings are directly connected to form aromatic layer sheets, and ether bonds often cross link the aromatic ring, so the decomposition of these oxygen-containing heterocycles and ether bonds can also lead to the fracture of aromatic layer sheets; that is, an increase of the 1 × 1 aromatic layer sheets. At the high-temperature stage, the large number of hydrocarbon products is mainly related to the removal of fatty side chains, and the large amount of methane generation is the result of the fracture of aromatic methyl or aromatic methyl ether bonds^34^. The removal of these bonds leads to the formation of new active sites, which is conducive to the polymerization of aromatic layers. After the removal of the oxygen-containing functional groups and the aliphatic hydrocarbons on the side chains, the aromatic fragments polymerized, resulting in a decrease in 1 × 1 aromatic fragments, and an increase in 2 × 2 and 3 × 3 aromatic fragments. The length of the aromatic layer was increased via condensation of the aromatic layer, showing that the 1 × 1 the aromatic layer significantly decreased, and that the 2 × 2, 3 × 3, 4 × 4, 5 × 5 and 6 × 6 aromatic layers increased to varying degrees (Fig. 3b).

### Orientation of the aromatic layer

**Figure 4:**
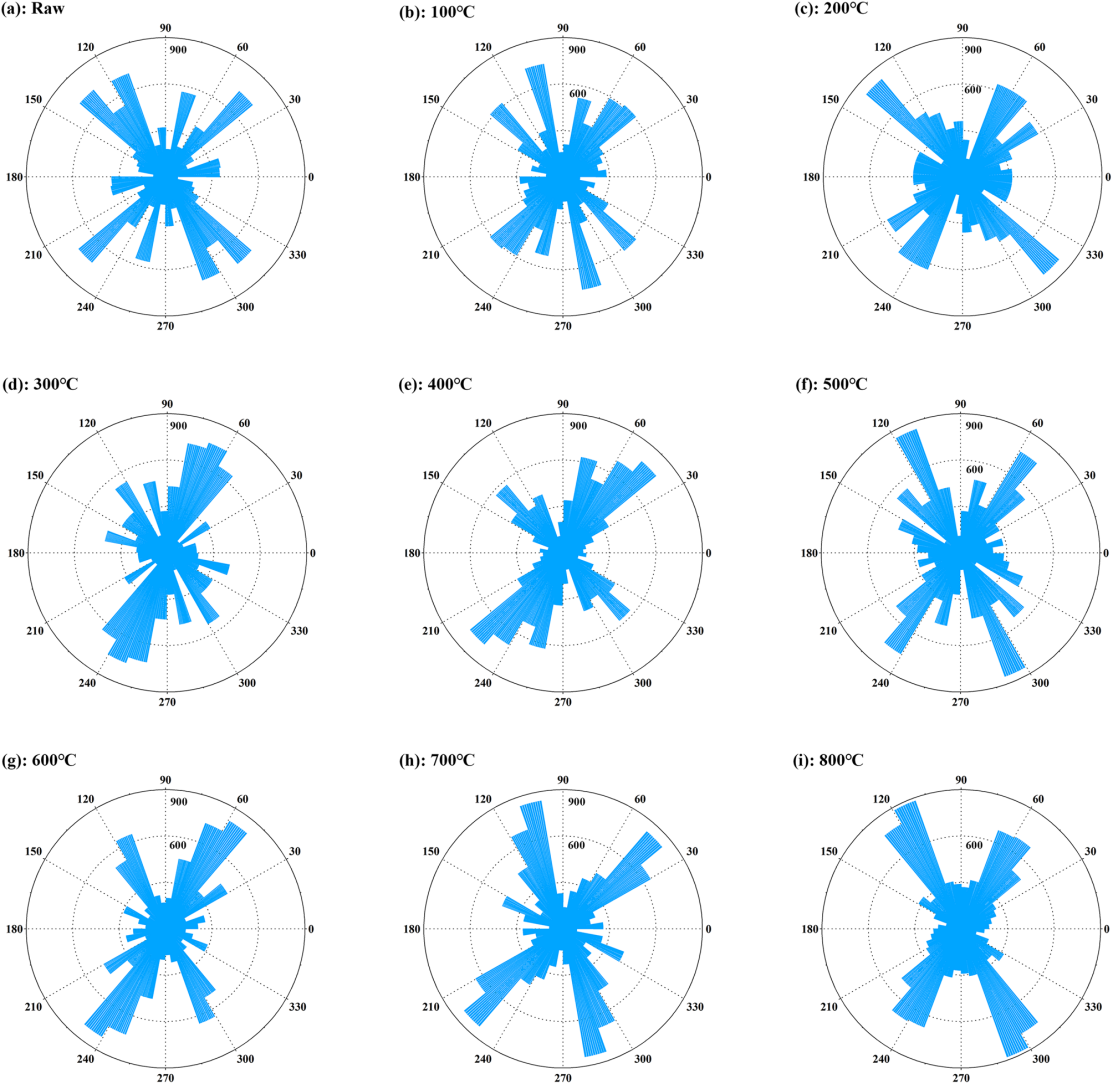
Orientation statistics of the lattice fringe of the heat treatments.

The orientation of the aromatic layers in coal tends to be orderly with the deepening of coalification. The degree of orientation can be used to measure the order degree of the aromatic layers. The coal structure is characterized by short range order and long range disorder. The order of the local structure cannot represent the entire coal structure. Therefore, the orientation distribution of all lattice fringes extracted from each residue sample was analysed. Take the slope of the line between the two endpoints of the lattice fringe as the orientation angle, convert it to 0°–360°, and count at 10° intervals (Fig. 4).

Figure 4 shows the orientation distribution of the aromatic layers in the heat-treated samples. The crystal lattice striations of the coal samples were mainly distributed in the four directions of 40°–70°, 110°–140°, 220°–250°, and 290°–320°. From low temperature to high temperature, the order of ambient temperature to 300 °C becomes worse, which is the result of the large-scale polymerization of aromatic lamellae generated in the early stage. During the polymerization process, the size of the middle lamellae increases and the position of the lamellae also rearranges. The lattice fringes at 400–600 °C were distributed in the main direction and their orientation range was reduced, indicating that the order of aromatic layers was enhanced. The reason might be that the smaller aromatic layers generated by the cracking of oxygen-containing heterocycles and ether bonds on the aromatic structure would move towards the direction of the lowest potential energy. From 700 to 800 °C, the fat structure connected to the aromatic structure cracks, and part of the aromatic system collapsed. The generated aromatic structure is rapidly condensed and rearranged after removing the heterogroups, resulting in enhanced order^35^.

Overall, the heat treatment changes the orientation of coal, but the main body orientation is consistent. From the coalification effect, as the heat treatment temperature increases, the lattice of orientation has a targeted trend. It can be seen from the ratio of the rose map (Fig. 4) that the striped area tends to 110°–120° and 50°–60° orientation. At the same time, it also proves that the coal sample may be low rank coal.

### Degree of the curve of the aromatic layer

**Figure 5:**
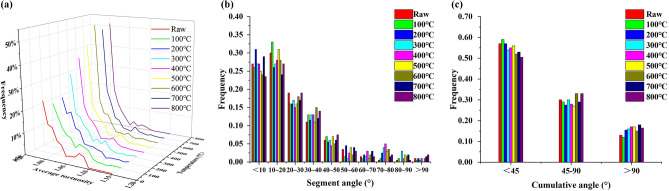
Degree of curve of lattice fringe of heat treatments. (**a**: Average tortuosity; **b**: Segment angle; **c**: Cumulative angle).

The average bending degree of the aromatic layer of the sample is between 1.0 and 1.2, and the proportion is greater than 90% (Fig. 5a). With an increasing temperature, the aromatic laminae with an average bending degree between 1.0 and 1.2 first decreased and then increased. From ambient temperature to 400 °C, due to the reduction of oxygen-containing heterocycles, the length of the aromatic layers decreased and the average curvature of aromatic layers decreased. The aromatic layers with an average curvature of 1.0–1.2 increased, while those with the average curvature of greater than 1.2 decreased. In the range of 400–600 °C, the smaller aromatic structural units were enriched due to the polymerization, and the average bending became larger. However, in the temperature range of 600–800 °C, affected by the enrichment of defect sites, the amount of aromatic layers that should have decreased (the average curvature is within 1.0–1.2) increased instead. This is because the amount of 1 × 1 aromatic layers decreased significantly while the amount of 2 × 2 aromatic layers increased. Due to the removal of a large number of fatty side chains and oxygen-containing functional groups, defect formation in this stage is weakened.

The distribution of fringe segmental angles during heat treatment is shown in Fig. 5b. The segmenting angles are mostly distributed from 0° to 20°, which reflects the good linearity of the lattice fringes of coal samples on the scale of their aggregation state. However, after heat treatment, the proportion of the fringe segment angle between 0° and 20° gradually decreases, which reflects that the decrease in bending due to heat treatment is consistent with the change in the average bending. The block angle at 20°–40° increases gradually, although after the heat treatment of stripes on the entire lattice fringe, the orientation is consistent, but does not reflect a good linearity on a single stripe, in which there are varying degrees of bend. A stripe may exist in the more non-six-membered ring (including a five-membered ring or a seven-membered ring). However, a piecewise analysis alone is not sufficient to quantify the curvature of the lattice fringes (it does not reveal which fringes cause bending).

Figure 5c shows the distribution of the accumulated fringe angles. Most of the curved fringe has low/moderate curvature. After heat treatment, the number of fringes with a cumulative angle < 45°obviously decreased. The cumulative angle is related to the length of the stripes, where the long stripes have more segmented angles. After high temperature treatment, the overall orientation of the coal sample fringes is good, but it shows a large bending of single fringes, where most of the bent fringes are more reflected in the short fringes. This is consistent with the conclusion obtained from the analysis of the piecewise angle distribution of the fringe^36^.

After heat treatment, aliphatic groups, aliphatic functional groups and side chains gradually fall off the thick ring of the aromatic core of coal sample, which also leads to a large difference in the orientations of the aromatic structure, which is reflected in the different orientation of the lattice fringes in each region, but the final trend is always aligned in the main direction. The fringes have good linearity at low temperature. However, their overall orientation is not good. After a high temperature treatment, the stripe, which overall has good orientation on a single stripe, has a larger bending, concrete embodiment in a long stripe with good linearity and a short stripe poor linearity (long streaks can represent most of the aromatic structure in coal, and short stripes may be part of the loss of fat structure, which can better explain the bending phenomenon). This reflects the complexity of coal aggregated structure^37^.

### Stack structure

**Figure 6:**
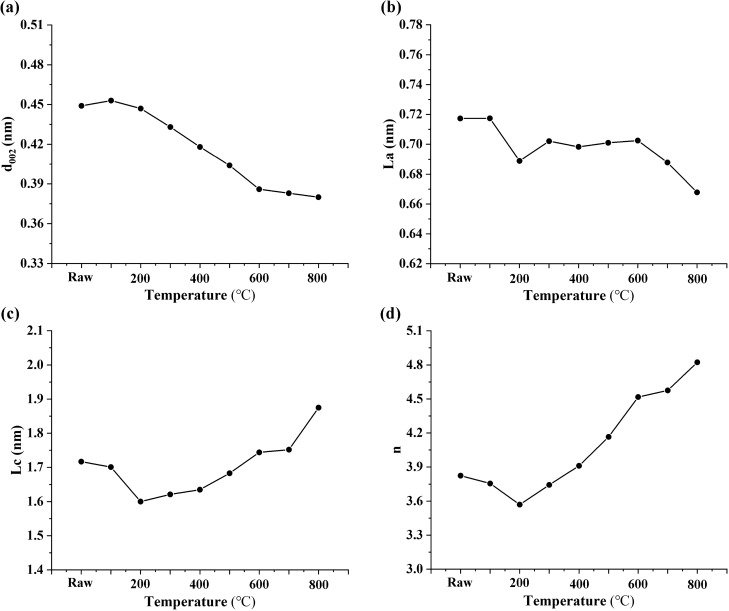
Changes in the stack structural parameters with heat treatment. (**a**: d_002_, **b**: La, **c**: Lc, **d**: n).

The average number of aromatic layers (n), average layer size (La), average interlayer spacing (d_002_) and average height of aromatic layers (Lc) for each stacking were obtained from HRTEM images. The HRTEM results showed (Fig. 6) that the microcrystalline parameters (d_002_, La, Lc and n) changed significantly when heated from ambient temperature to 800 °C, which was caused by changes in the chemical structure during the heat treatment.

Figure 6a, shows a clear relationship between the temperature and the value of the average layer spacing. The average value of d_002_ decreases with an increasing temperature. That is, with an increase in graphitization degree, the distance between aromatic layers in coal decreases gradually. The limit value is 0.3354 nm for the ideal graphite layer spacing. In this study, the d_002_ value drops from 0.45 to 3.8, indicating that 800 °C is not the temperature at which the structure will not change during coal heat treatment. Specifically, the value of d_002_ is almost constant in the range of ambient to 200 °C, significantly decreases in the range of 300–600 °C, and slightly decreases in the range of 600–800 °C. Although the average layer spacing of the aromatic layers decreases, the average stacking height (Lc) and the average number of layers (n) increase with an increasing temperature (Fig. 6c,d). The three parameters were all measured from the orderly part of the stacking. The steady variation in the parameters indicated that the aromatic layers continued to move towards compact stacking during the thermal simulation process. The trends of Lc and n values of the coal samples are similar. The variation in Lc ranged from 1.6 to 1.88 nm, and the variation in n was ranged 3.7–4.8. The number of stacking layers increases obviously with a higher plasticity of the coal sample, which increases the stacking height. The average layer size (La) obtained by the lattice fringe length decreases with an increasing temperature and ranges from 0.67 to 0.71 nm (Fig. 6b).

From an ambient temperature to 300 °C, the average values of La, Lc and n of coal samples decreased, which may be the evolution of gas products in the pyrolysis process. Only small amounts of CO_2_ and H_2_O were generated within this temperature range. Along with the deformation of various side chains and functional groups in the chemical structure, it shows poor orientation in the chemical structure. From 300 to 500 °C, d_002_ values decrease significantly, while the La, Lc, and n values increase. The main reaction in this temperature range is the breaking of amorphous materials weakly bound to the aromatic layer, such as aliphatic side chains and oxygen-containing functional groups. Being rich in aliphatic groups, especially CH_2_, they release a large number of volatile substances (such as CH_4_, CO_2_, H_2_O, etc.). The removal of CH_4_ is attributed to the breakdown of aliphatic side chains. The production of CO_2_ and H_2_O mainly comes from oxygen-containing groups^38^. In addition, in the range of 400–500 °C, volatiles are released and a large number of free radicals are generated, accompanied by strong thermal decomposition. The aliphatic groups are decomposed and some aromatic layers are concentrated, resulting in more stacking observed in the HRTEM images, indicating that the development of microcrystalline units is also improved. When the temperature reaches 600 °C, d_002_ decreases, and La and Lc increase significantly, indicating that the aromatic hydrocarbons change greatly. Some gases are then produced (H_2_, CH_4_, etc.). The structure is rearranged and enhanced to form a perfect crystal structure and increase the order of the crystal structure. By coalescence of the adjacent layers, the layer grows and results in a wide distribution of molecular sizes. The decrease in La is caused by the production of H_2_, which mainly comes from the ring dehydrogenation of multinuclear condensation at high temperature^39^.

### Utramicropores

**Figure 7:**
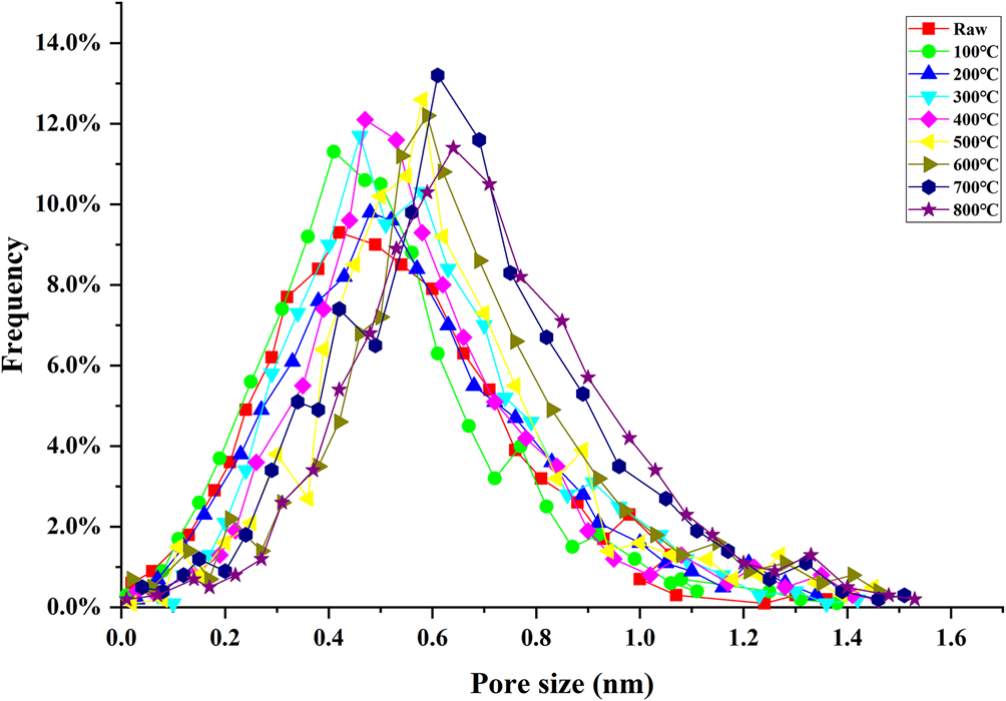
Pore size distribution of the heat treatments.

The ultramicropores size of the coal samples extracted by HRTEM ranges from 0 to 1.5 nm, and the pore size is mainly concentrated from approximately 0.4–0.7 nm, with more micropores developed (Fig. 7). After heat treatment, the peak value and the peak height of pore diameter of the coal sample obviously increase^40^.

The micropores of raw coal samples are mostly in the closed state, the mesopores and macropores have good connectivity, and the water exists in a free water stste. After thermal driving at 100 °C, the closed hole of coal is opened, resulting in water loss in the closed hole; however, it is still not enough to change the pore structure of coal. With the increase of temperature, the expansion effect caused by thermal stress changes the pore structure. The high temperature thermal drive of 200 °C starts to increase the pores inside the coal body, making the smaller pores connect to the larger pores, and the larger pores transform to the smaller cracks. As a result, the average size of the coal holes increases as a whole, but it has little influence on the ultramicropores. When the temperature is 300–600 °C, the coal sample chemical composition is constantly changing, and the coal's ultramicropores change greatly. The organic and inorganic components in coal will undergo physical and chemical changes after being heated, and the removal of water and free alkyl-hydrogen, nitrogen and C–O compounds, depolymerization and condensation reactions will break the side chains in coal. The coal sample begins to be pyrolyzed, the aromatic ring and thick ring begin to decompose, side chain breaks, gas is formed, the gas gradually spills out, the coal skeleton gradually becomes empty, and the characteristics of ultramicropores change dramatically. Especially from 500 to 600 °C, the skeleton of coal continues to undergo depolymerization reaction, the ultramicropores are interconnected, the number of ultramicorpores decreases greatly, and the volume increases sharply. From 700 to 800 °C, the polycondensation reaction occurs in the coal sample, a large number of fatty side chains and oxygen-containing functional groups are removed, and the change in ultramicropore characteristics slows down.

### Relevant analysis of the activation energy and HRTEM parameters

**Figure 8:**
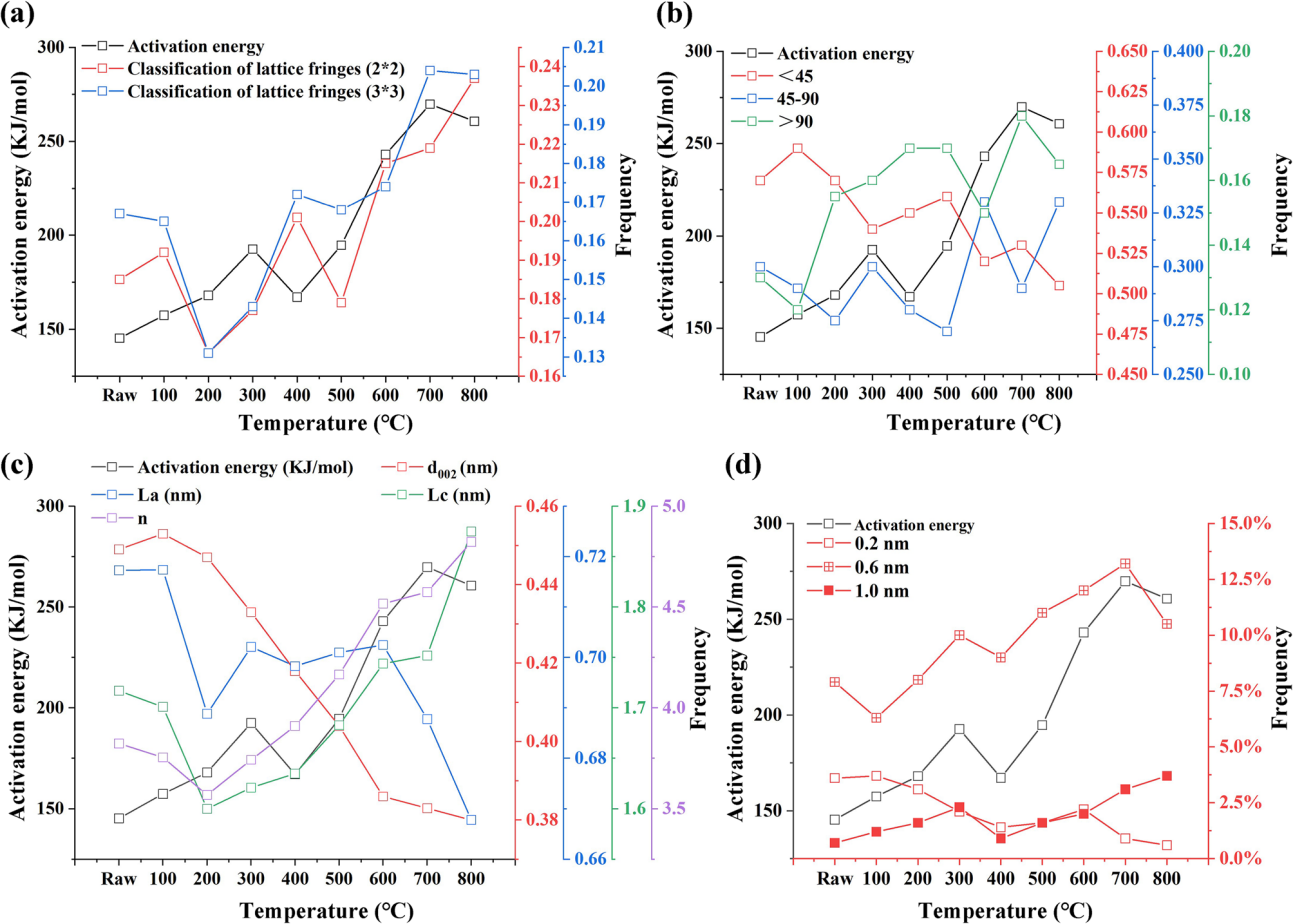
Activation energy of heat treatments. (**a**: activation energy values with classification of lattice fringes, **b**: activation energy values with the cumulative angle, **c**: activation energy values with the stack structure, **d**: activation energy values with the pore size).

According to the analysis of the HRTEM parameters and activation energy, some parameters are consistent with the change trend of the activation energy (Fig. 8).

It was determined that the content of the aromatic layer and reaction activation energy (Fig. 8a) showed similar trends, especially in the direction of the 2 × 2 and 3 × 3 layers. In addition, there is a complex relationship between the activation energy and the accumulation angle (Fig. 8b). When the accumulation Angle is less than 45°, they show an opposite trend, while when the accumulation angle is between 45° and 90°, they show the same trend, and when the accumulation angle is greater than 90°, the trend of the two is not obvious. By comparing the change trend of the activation energy and the stack structure parameters (Fig. 8c), an obvious temperature change node (300 °C) can be found. At ambient temperature to 300 °C, all stack structure parameters show a downward trend, during which the coal sample rapidly loses water and combines with air. At 300 °C, the activation energy showed the same upwards trend as the effective stacking height (Lc) and the stacking layer number (n), but the opposite trend as the aromatic lamellae spacing (D_002_). In the process of heat treatment, the frequency and activation energy values were compared when the size of the ultramicropores was 0.2 nm, 0.6 nm and 1.0 nm, and the change trend of the activation energy and the frequency of ultrafine pores at 0.6 nm was the same (Fig. 8d).

In general, the HRTEM characteristics are significantly different from those of raw coal when the temperature is above 300 °C. The changes in the content and activation energy of the aromatic layer (2 × 2 to 8 × 8) increased slowly at first, and then increased rapidly at 300 °C. When the number of oxygen-containing functional groups decreases, the number of aromatic layers with high ring numbers decreases, that is, the oxygen removed in the evolution process is the oxygen-containing functional groups in the aromatic layers with high ring numbers. When the oxygen-containing functional groups are reduced, the starting temperature of pyrolysis decreases, that is, the thermal stability decreases, and it is easy to remove. When the number of aromatic layers with high ring number decreases, the bond energy decreases and the temperature required for pyrolysis decreases. In summary, when the number of aromatic layers with high ring number is reduced, the breakage of cross-linking bonds leads to a reduction of defects between aromatic layers, which may be caused by the defects of oxygen-containing functional groups. With a decrease in the oxygen-containing functional groups, the thermal stability is poor, and the temperature required for pyrolysis is reduced, and vice versa^41^.

## Conclusion

By analysing HRTEM characteristics of coal samples after heat treatment, the following conclusions were obtained:

When the temperature reaches 400 °C, HRTEM characteristics are obviously different from those of raw coal. From 300 to 500 °C, the number of 1 × 1 aromatic layers increased, the number of larger aromatic layers decreased, the number of aromatic layers with curvature between 1.0 and 1.2 increased, the number of aromatic layers with greater curvature decreased, the distance between aromatic layers decreased, the stacking height increased, and the order of aromatic layers was enhanced. These changes were related to the decomposition of oxygen-containing functional groups. From 500 to 600 °C, 1 × 1 aromatic sheets decreased, larger aromatic sheets increased, and those with bending degree between 1.0 and 1.2 decreased, while those with larger bending degree increased. The spacing of the aromatic sheets decreased, the stacking height increased, and the order of aromatic sheets improved better. This was related to the fracture of aliphatic hydrocarbons in coal and the condensation of small aromatic sheets. From 600 to 800 °C, 1 × 1 aromatic layers decreased, larger size aromatic layers increased, the curvature of 1.0–1.2 aromatic layers decreased, larger curvature of aromatic layers increased, the aromatic layer spacing slightly increased, stacking height significantly increased, and the order of aromatic layers became worse. These changes were related to the condensation polymerization reaction of small aromatic layers.

In addition, there is correlation between the activation energy and the coal aggregation state and ultramicropores. Among them, the content of the aromatic layer and the activation energy of the reaction showed similar trends, while the activation energy and the cumulative angle of different degrees showed different laws. It is worth mentioning that the activation energy and the stack structure parameters have a sharp transition at 300 °C, and the activation energy has the same increasing trend with the effective stacking height (Lc) and stacking layer number (n), but the opposite trend with an increasing trend of aromatic layer spacing (d_002_). Moreover, when the ultramicropore size is 0.6 nm, the ultramicropore size after heat treatment is closely related to the activation energy. However, current experiments cannot explain the underlying causes of these relationships.

The fat functional groups and aromatic structure in coal also affect the distribution of pores (< 2 nm) in coal. Non-six-membered rings and lattice defects lead to fringe bending, the distribution of fatty structures affects the fringe orientation, and the relationship between pore and molecular structure does not exist independently. Different temperature treatments have different fringe distributions, aliphatic chains and pore size distributions, which results in different aggregation states in coal, reflecting the heterogeneity of coal. Aggregated states in coal are macromolecular groups formed between different aromatic structures, fat structures and other molecules, and formed by the interaction of defects and pores within the molecular groups. The structural differences of different treatments reflect the interaction results of different macromolecules in coal.
